# Overexpression of IGFBP2 mRNA predicts poor survival in patients with glioblastoma

**DOI:** 10.1042/BSR20190045

**Published:** 2019-06-14

**Authors:** Qing Yuan, Hong-Qing Cai, Yi Zhong, Min-Jie Zhang, Zhi-Jian Cheng, Jia-Jie Hao, Ming-Rong Wang, Jing-Hai Wan

**Affiliations:** 1 Department of Neurosurgery, The Second Affiliated Hospital of Anhui Medical University, Hefei, 230601, P.R. China; 2 Department of Neurosurgery, National Cancer Center/National Clinical Research Center for Cancer/Cancer Hospital, Chinese Academy of Medical Sciences and Peking Union Medical College, Beijing 100021, P.R. China; 3 State Key Laboratory of Molecular Oncology, National Cancer Center/Cancer Hospital, Chinese Academy of Medical Sciences and Peking Union Medical College, Beijing 100021, P.R. China

**Keywords:** Glioblastoma, IGFBP2, RNA In situ hybridization (RISH), TERT

## Abstract

The prognosis of patients with glioblastoma (GBM) is dismal. It has been reported that Insulin-like growth factor (IGF) binding protein 2 (IGFBP2) is associated with the mobility and invasion of tumor cells. We investigated the expression of IGFBP2 mRNA in GBMs and its clinical relevance, using tissue microarrays and RNAscope *in situ* hybridization in 180 GBMs and 13 normal or edematous tissues. The correlations between the expression and clinical pathological parameters as well as some other biomarkers were analyzed. Overexpression of IGFBP2 mRNA was observed in 23.9% of tumors tested. No expression of IGFBP2 mRNA was detected in normal or edematous tissues. Kaplan–Meier survival analysis showed that the survival time of all the patients with high IGFBP2 tumors had shorter survival than those with low IGFBP2 (*P*<0.01). Univariate regression and multivariate regression both indicated that the expression of IGFBP2 transcript level was an independent prognostic factor (*P*=0.008 and 0.007, respectively). Furthermore, expression of IGFBP2 mRNA was related to the occurrence of isocitrate dehydrogenase 1 (IDH1) mutation, high heat shock protein 27 (Hsp27) expression and telomerase reverse transcriptase (TERT) promoter mutation (TERTp^+^) (*P*=0.013, 0.015 and 0.016, respectively), and patients with TERTp^+^/IGFBP2^high^ showed the shortest survival. In conclusion, IGFBP2 mRNA expression status is an independent prognostic biomarker in GBMs, and the combination of IGFBP2 mRNA and TERTp status might serve as a prognostic indicator in patients with GBM.

## Introduction

Glioblastomas (GBMs), as WHO IV gliomas, are the most aggressive primary brain malignancy in adults [1]. Biologically, GBM cells present an uncontrolled cellular proliferation, diffuse infiltration, resistance to apoptosis, and genomic instability [2]. Besides, great anatomical and molecular heterogeneity within GBM bulk tumor could result in resistance to drug and radiation [3]. Previous clinical studies demonstrated that concurrent radio-chemotherapy and followed chemotherapy after surgical removal of the tumor has greater clinical efficacy than traditional therapy. Thus, the therapeutic regimen has become the standard treatment for GBMs. However, patients suffering from GBMs still have a dismal prognosis with a median overall survival (OS) of 15–17 months in clinical management [4–6]. Thus, an understanding of the molecular alterations of gliomas is critical for improving clinical management for patients with this disease.

Insulin-like growth factor (IGF) binding protein 2 (IGFBP2) is a member of the secreted IGFBP family that functions by interacting with circulating IGFs to modulate IGF-mediated signaling [7]. It has been observed that overexpression of IGFBP2 promoted an up-regulation of migration and invasion [8–11]. Increased IGFBP2 expression has conferred chemotherapy resistance in multiple types of malignancies including breast cancer [12–14], colon cancer [15] and lung cancer [16–18]. In GBM, IGFBP2 overexpression significantly increased the invasive capability of tumor cells [11,19]. RNA sequencing analysis showed that high IGFBP2 transcript level is associated with malignant clinical features of GBMs [20]. However, the studies of IGFBP2 transcript expression in GBM specimen detected by *in situ* method are limited.

In the present study, we used RNAscope probe to detect the expression of IGFBP2 mRNA in 180 GBMs. We found that IGFBP2 mRNA showed higher expression in a subset of GBM tissues than normal or edematous tissues. IGFBP2 overexpression was an independently unfavorable prognostic factor. The combination of IGFBP2 expression and telomerase reverse transcriptase (TERT) promoter (TERTp) status could better predict the prognosis for patients with GBM.

## Materials and methods

### Patients and tissue samples

One hundred and eighty GBMs and thirteen paired normal or edematous brain tissue specimens were procured from the Department of Neurosurgery at the National Cancer Center (NCC)/Cancer Hospital of Chinese Academy of Medical Sciences. Normal or edematous tissues were acquired by two ways: normal superficial brain tissues resected for getting to deep-seated glioma and edematous tissues around high-grade glioma. All the specimens used in the present study were residual tissues collected after diagnostic sampling. No patients received neoadjuvant treatment before neurosurgery, and signed separate informed consent forms for the sample collection and molecular analysis. This research has been carried out in accordance with the World Medical Association Declaration of Helsinki. Primary tumor regions from freshly excised research tumor tissues were sampled by experienced pathologists, and immediately stored at −80°C until used. Data were recorded concerning the clinical/pathological parameters of each tumor, including age/sex of patients, pathological grade and treatment information (Table 1). The present study was approved by the Ethics Committee of the Cancer Hospital, Chinese Academy of Medical Sciences (Number NCC2014G-12).

**Table 1:** Baseline information of 180 GBMs by IGFBP2 mRNA *in situ* hybridization

Variables	No	IGFBP2 (high)	IGFBP2 (low)^1^	χ^2^	*P*-value
Gender				0.201	0.654
Male	112	28 (25.0%)	84 (75.0%)		
Female	68	15 (22.1%)	53 (77.9%)		
Age (years)				4.435	0.035
≤50	88	15 (17.0%)	73 (83.0%)		
>50	92	28 (30.4%)	64 (69.6%)		
KPS^2^				3.464	0.063
≤60	32	9 (28.1%)	23 (71.9%)		
>60	44	5 (11.4%)	39 (88.6%)		
NA^3^	104	29 (27.9%)	75 (72.1%)		
Radiotherapy				3.212	0.073
Yes	112	32 (28.6%)	80 (71.4%)		
No	66	11 (16.7%)	55 (83.3%)		
NA	2	0 (0%)	2 (100%)		
Chemotherapy				0.054	0.816
Yes	151	36 (23.8%)	115 (76.2%)		
No	27	7 (26.0%)	20 (74.0%)		
NA	2	0 (0%)	2 (100%)		
Pathology^4^				10.092	0.001
pGBM	140	41 (29.3%)	99 (70.7%)		
sGBM	40	2 (2.5%)	38 (97.5%)		

1 IGFBP2 (high), high expression of IGFBP2 mRNA in GBM; IGFBP2 (low), low expression of IGFBP2 mRNA in GBM.

2 KPS, Karnofsky score. It is clinically used to evaluate patients’ functional status.

3 NA, not available (the same below).

4 pGBM, primary GBM; sGBM, secondary GBM.

### Tissue microarray and IGFBP2 mRNA RNAscope assay

The tissues were fixed in formalin and embedded in paraffin. A section of 4 μm was made from the paraffin donor block, and stained with Hematoxylin and Eosin (H&E) to define tumor and normal or edematous regions. For 13 tumors with matched normal or edematous tissues, tumor tissues were in triplicate and normal or edematous tissue was in duplicate. For the others, five tumor tissue cores were taken from the primary block. The resulting blocks were cut into 5-µm sections.

RNAscope probes were purchased from Advanced Cell Diagnostics (ACD, Newark, LA). The probe of human gene IGF binding protein 2 (IGFBP2) was against the transcript NM_000597.2, bp 490 – 1423, ACD#313061, and those of Ubiquitin C (UBC, NM_021009, bp 342–1503, ACD#310041) and DapB (ACD#310043) were used as positive and negative controls, respectively. The RNA *in situ* hybridization (RISH) was done as protocol described. RISH slides were scanned using a NanoZoomer (Hamamatsu, Japan) high-resolution scanner, and the results were scored blindly with no information on clinical data. IGFBP2 expression levels were determined on the basis of staining intensity and the percentage of positive cells. Staining intensity was rated as 0 (negative), 1 (weakly positive), 2 (moderately positive), and 3 (strongly positive). The percentage of positive cells was graded as 0 (0%), 1 (1–20%), 2 (21–50%), and 3 (51–100%). The average score of tumor cell staining intensity multiplied by the score of the percentage of positive cells represented the final score of the specimens. A final score of ≥3 was considered as high expression for each case.

### Immunohistochemistry

Immunohistochemistry was performed as described previously [21]. The following antibodies were used: anti-heat shock protein 27 (Hsp27) antibody (1:500, 50353, CST), anti-IDH1^R132H^ antibody (working solution, ZM0447, ZSGB-BIO). The immuno-scores for these antibodies were evaluated as previously described [22].

### DNA extraction and Sanger sequencing

Genomic DNA was extracted from consecutive GBM FFPE sections of 10 μm using the QIAamp DNA mini kit (Qiagen). DNAs were quantitated using Nanodrop (Thermo Fisher Scientific). Nested PCR was performed to amplify the target region of the TERT promoter containing C228 and C250 (chr5: 1295228; chr5: 1295250, respectively; hg19). Primer sequences for the first PCR were 5′-GTCCTGCCCCTTCACCTT-3′ (forward) and 5′-GCACCTCGCGGTAGTGG-3′ (reverse). 5′-TTCCAGCTCCGCCTCCT-3′ (forward) and 5′-GCGCTGCCTGAAACTCG-3′ (reverse) were for the second PCR. PCR procedures were performed using a C1000 thermal cycler (Bio-Rad) with an initial denaturing step at 95°C for 5 min, followed by 30 cycles of denaturation at 96°C for 15 s, annealing at 60°C for 30 s, extension at 72°C for 30 s, and a final extension at 72°C for 10 min. PCR products were subjected to direct sequencing on an ABI3730 PRISM DNA sequencer of Tianyi Huiyuan Bioscience & Technology Inc (Beijing, China).

### Statistical analysis

Significant differences between two groups were determined by the Mann–Whitney U test. X-tile software (Version 3.6.1, Yale University, U.S.A.) was used to ascertain the optimal cut-off points for survival analysis. The χ^2^ test was used to assess the relationship between molecular alterations and clinico-pathological parameters. OS curves were plotted according to the Kaplan–Meier method, with the log-rank test applied for comparison. Multiple Cox proportional hazard model was used to predict independent prognostic factors. All statistical analyses were performed using both IBM SPSS Statistics 21.0 and GraphPad Prism 5.0. *P*-value less than 0.05 was considered statistically significant. All tests were two-sided.

## Results

### Overexpression of IGFBP2 mRNA in GBMs

**Figure 1:**
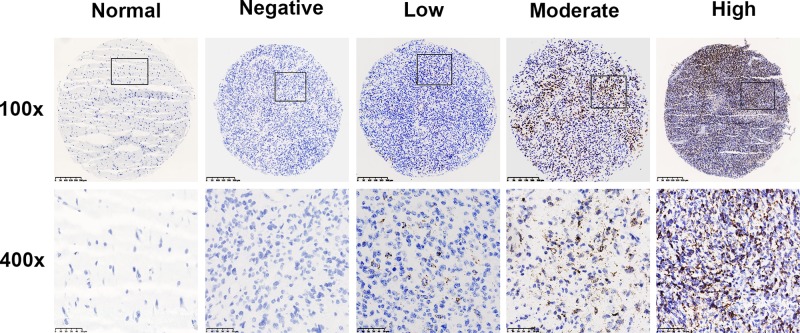
Representative RISH images of negative, low, moderate, and high IGFBP2 mRNA expression (100× and 400×)

All of the GBMs had positive RISH signals for UBC, but none had signals for DapB (Supplementary Figure S1A). RISH detection showed that IGFBP2 was of high expression in 23.9% (42/180) of the tested GBMs. The positive signal anatomically located in cellular tumor area, as well as area around necrosis (Supplementary Figure S1B). All the morphologically normal or edematous tissues presented totally negative IGFBP2 mRNA (Figure 1). We examined the relationship between IGFBP2 transcript level and clinic-pathological features. IGFBP2 mRNA was significantly higher expressed in primary than that in secondary GBMs (29.3 vs 2.5%, *P*=0.001). Besides, elderly patients (>50 years) tended to present with a higher positive rate of IGFBP2 mRNA than younger patients (≤50 years) (*P*=0.035) (Table 1).

### IGFBP2 mRNA expression associated with other biomarkers of GBMs

**Figure 2:**
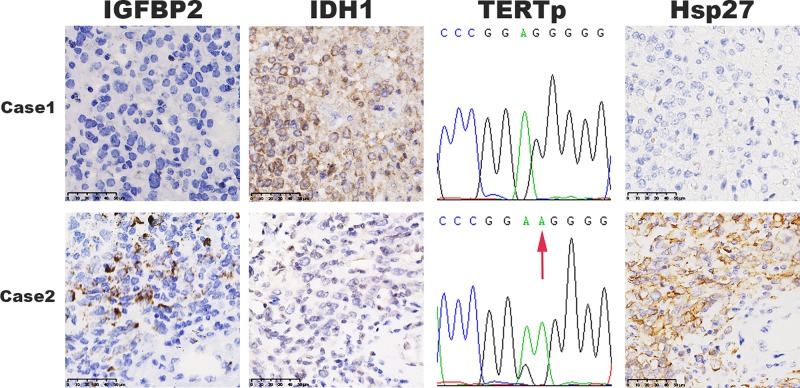
Representative multi-marker detection results Case 1, IGFBP2^low^/IDH1^+^/TERTp^−^/Hsp27^low^; Case 2, IGFBP2^high^/IDH1^−^/TERTp^+^/Hsp27^high^.

For the same cases, we previously reported high expression of Hsp27 protein and isocitrate dehydrogenase 1 (IDH1) mutation in tumors [22]. In the present study, we further investigated TERTp mutation, and simultaneously analyzed the relationship between IGFBP2 mRNA expression and the alterations of TERTp, Hsp27 and IDH1 (Figure 2). In the 180 tumors tested, TERT promoter presented a mutation in 55.3% cases (30.3% as C250T and 69.7% as C228T). We found that IGFBP2 expression was positively correlated with the expression of Hsp27, TERT promoter mutation (*P*=0.015 and 0.016), but negatively correlated with IDH1 mutation (*P*=0.013, Table 2).

**Table 2:** Relationships between IGFBP2 and other biomarkers in GBMs

Biomarker	Status	IGFBP2		χ^2^	*P*
		High	Low		
IDH1	Mutation	7	50	6.182	0.013
	Wlid-type	36	87		
Hsp27	High	19	34	5.910	0.015
	Low	24	103		
TERTp	Mutation	30	69	5.770	0.016
	Wlid-type	12	68		

### IGFBP2 mRNA and TERT promoter mutation correlated with survival of GBM patients

**Figure 3:**
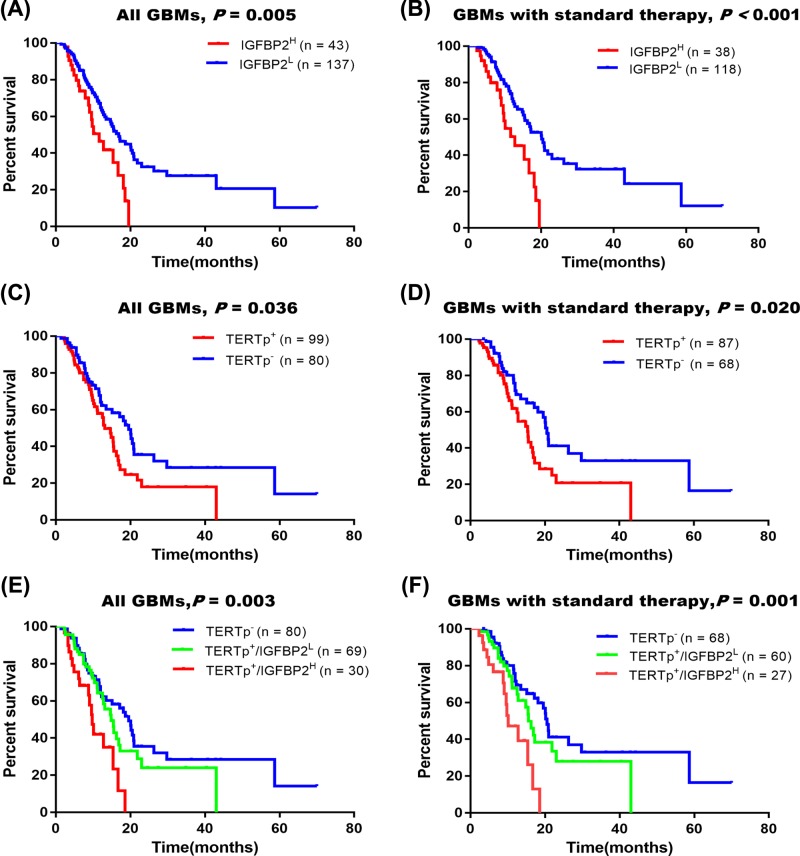
The prognostic value of IGFBP2 mRNA and TERT promoter mutation (**A**–**D**) Overexpression of IGFBP2 mRNA (IGFBP2^H^) and TERT promoter mutation (TERTp^+^) predicted shorter survival of patients with GBM. (**E,F**) Multi-markers detection can stratify the patients into three groups with significantly different survival times, in which the patients with IGFBP2^H^/TERTp^+^ had a shorter survival.

By the analysis of IGFBP2 mRNA expression and OS of the patients, we observed that the high expression of IGFBP2 mRNA predicts a shorter survival than the low IGFBP2 (mOS = 11.6 vs 16.9 months, *P*=0.0046, Figure 3A). When we exclude the patients who did not receive standard therapy, predicting prognosis of high IGFBP2 expression was more significant (*P*=0.0008, Figure 3B). Univariate Cox regression model showed that radiotherapy, chemotherapy, and IGFBP2 mRNA expression were related to survival time of the patients. And multivariate Cox regression analysis demonstrated that IGFBP2 mRNA expression was an independent shorter survival indicator (Table 3).

**Table 3:** Univariate and multivariate regression analyses of factors associated with disease- specific survival in GBMs

Variable	Univariate regression		Multivariate regression	
	HR (95% CI)^1^	*P*-value	HR (95% CI)	*P*-value
Age (≤50, >50)	1.327 (0.867–2.032)	0.193	-	-
Gender (male, female)	0.896 (0.587–1.367)	0.609	-	-
KPS (≤60, >60)	0.691 (0.373–1.282)	0.242	-	-
Pathology (pGBM, sGBM)	0.796 (0.485–1.307)	0.368	-	-
Radiotherapy (Y, N)^2^	0.306 (0.198–0.474)	<0.001	0.293 (0.184–0.467)	<0.001
Chemotherapy (Y, N)	0.232 (0.134–0.404)	<0.001	0.382 (0.213–0.684)	0.001
IDH1 (mutation, wild-type)	0.526 (0.327–0.844)	0.008	0.514 (0.316–0.837)	0.007
IGFBP2 (high, low)	2.004 (1.228–3.272)	0.005	2.356 (1.424–3.899)	0.001

1 CI, confidence interval; HR, hazard ratio.

2 N, no; Y, yes.

Also, our results showed that the patients with TERT promoter mutation exhibited significantly poorer survival than those with wild-type TERTp (*P*=0.036, Figure 3C). The results were similar when excluded patients who did not receive chemotherapy and/or chemotherapy after operation (*P*=0.020, Figure 3D).

### Overexpression of IGFBP2 mRNA stratifies patients by combining TERT promoter mutation

By combining IGFBP2 mRNA expression and TERT promoter status, survival analysis showed that GBM patients harboring TERTp wild-type (TERTp^−^) had the longest survival time (mOS = 19.6 months). In patients with TERTp mutation (TERTp^+^), the difference of IGFBP2 mRNA expression divides the patients into two subgroups: low IGFBP2 presented a longer survival, and those with both TERTp mutation (TERTp^+^) and high IGFBP2 (IGFBP2^high^) the shortest (mOS = 14.8 vs 9.8 months, *P*<0.001, Figure 3E). In addition, in the patients who had received postoperative adjuvant therapy, TERTp^+^/IGFBP2^high^ and TERTp^+^/IGFBP2^low^ presented a median survival of 10.2 and 15.6 months, respectively (*P*=0.001, Figure 3F).

## Discussion

Previous studies [20] and the present study all demonstrated that IGFBP2 mRNA is highly expressed in GBM and strongly related to clinical parameters of patients, which suggested that IGFBP2 might be involved in maintenance of malignant phenotypes in GBM cells. Additionally, we observed obvious difference of IGFBP2 mRNA expression between primary and secondary GBM. The fact that IGFBP2 mRNA predominantly expressed in primary GBM suggesting that IGFBP2 may participate in the origin of GBM.

It has been reported that IGFBP2 plays an important role in immunologic processes of GBM, and may become a potential target for immunotherapy in GBM [20]. In the present study, we investigated the expression levels and clinical significance of IGFBP2 mRNA in 180 GBMs. We observed a high expression rate of 23.9% in the tested tumors. However, the high expression rate was 41.3% in the data of the Chinese Glioma Genome Atlas (CGGA), and 80.6% in another report by Zheng et al. [23]. One of the reasons for so large difference might be from different detection methods and thresholds of high expression. In the data of CGGA and Zheng et al.’s [23] research, IGFBP2 mRNA was measured by transcriptome sequencing technique and Quantitative Reverse Transcription PCR (qRT-PCR). In the present study, we used RISH, which holds significant promise as a new platform for developing and implementing RNA-based molecular diagnostics. Also, different standards for high expression were used in these studies. And we considered the final score ≥ 3 as high expression, which is relatively high and appropriate for clinical practice in the future. Besides, the RISH results are location-visible and therefore more accurate than other methods to determine the expression of tumor cells.

IGFBP2 expression in the tumor has been proved in many researches that was associated with a variety of pathological conditions, including hypoxia [24] and regeneration [25]. In the present study, we founded IGFBP2^high^ cells accumulate in the immediate vicinity of focal necrosis of some tumors. The result suggested that IGFBP2 might play a role in hypoxia related pathways. Further studies we will make to demonstrate this hypothesis.

In the present study, we simultaneously investigated other candidate biomarkers of GBMs. In 2008, whole-genome analysis led to the discovery of recurrent mutations of IDH1 in secondary (those progressing from lower grade gliomas) rather than in primary GBMs [26]. We previously observed that Hsp27 and p-Hsp27 are predominately expressed in WHO grade III/IV astrocytic gliomas. Strong correlation between the expression of IGFBP2 mRNA and these biomarkers in GBM suggested that IGFBP2 may play an important role in the development or progression of glioma. Interestingly, our analysis showed a 16.9-month median OS for the patients with low IGFBP2 mRNA levels, but 11.6 months for those with high IGFBP2. By combining IGFBP2 mRNA expression with TERT promoter mutation, we divided that GBM patients into different subgroups. We observed that the patients with TERTp^+^/IGFBP2^high^ presented the shortest survival. Therefore, RISH results of IGFBP2 can predict the survival for the patients with TERT promoter mutation, including those who had received postoperative adjuvant therapy (chemotherapy and/or radiotherapy).

In conclusion, the present study revealed that IGFBP2 mRNA is an independent prognostic biomarker in GBMs. Together with TERT status, IGFBP2 mRNA might serve as a potential prognostic indicator in patients with GBM.

## Abbreviations

ACD: Advanced Cell Diagnostics
CGGA: Chinese Glioma Genome Atlas
GBM: glioblastoma
Hsp27: heat shock protein 27
IDH1: isocitrate dehydrogenase 1
IGF: insulin-like growth factor
IGFBP2: IGF binding protein 2
OS: overall survival
RISH: RNA *in situ* hybridization
TERTp: telomerase reverse transcriptase promoter
UBC: ubiquitin C
